# Genetic Variants of DNA Repair Genes as Predictors of Radiation-Induced Subcutaneous Fibrosis in Oropharyngeal Carcinoma

**DOI:** 10.3389/fonc.2021.652049

**Published:** 2021-05-17

**Authors:** Ankita Gupta, Don Mathew, Shabir Ahmad Bhat, Sushmita Ghoshal, Arnab Pal

**Affiliations:** ^1^ Department of Radiotherapy, Post Graduate Institute of Medical Education and Research, Chandigarh, India; ^2^ Department of Biochemistry, Post Graduate Institute of Medical Education and Research, Chandigarh, India

**Keywords:** single nucleotide polymorphisms (SNPs), subcutaneous fibrosis, radiation-induced toxicity, DNA repair genes, oropharyngeal carcinoma

## Abstract

**Purpose:**

To investigate the impact of genetic variants of DNA repair and pro-fibrotic pathway genes on the severity of radiation-induced subcutaneous fibrosis in patients of oropharyngeal carcinoma treated with radical radiotherapy.

**Materials and Methods:**

Patients of newly diagnosed squamous cell carcinoma of oropharynx being treated with two-dimensional radical radiotherapy were enrolled in the study. Patients who had undergone surgery or were receiving concurrent chemotherapy were excluded. Patients were followed up at 6 weeks post completion of radiotherapy and every 3 months thereafter for a median of 16 months. Subcutaneous fibrosis was graded according to the Radiation Therapy Oncology Group (RTOG) and the European Organization for Research and Treatment of Cancer (EORTC) grading system and the maximum grade was recorded over the length of the patient’s follow-up. Patients with severe fibrosis (≥G3), were compared to patients with minor (≤G2) fibrotic reactions. Eight single nucleotide polymorphisms of 7 DNA repair genes and 2 polymorphisms of a single pro-fibrotic pathway gene were analyzed by Polymerase Chain Reaction and Restriction Fragment Length Polymorphism and were correlated with the severity of subcutaneous fibrosis.

**Results:**

179 patients were included in the analysis. Subcutaneous fibrosis was seen in 168 (93.9%) patients. 36 (20.1%) patients had severe (grade 3) fibrosis. On multivariate logistic regression analysis, Homozygous CC genotype of XRCC3 (722C>T, rs861539) (p=0.013*, OR 2.350, 95% CI 1.089-5.382), Homozygous AA genotype of ERCC4 Ex8 (1244G>A, rs1800067) (p=0.001**, OR 11.626, 95% CI 2.490-275.901) and Homozygous TT genotype of XRCC5 (1401G>T, rs828907) (p=0.020*, OR 2.188, 95% CI 1.652-7.334) were found to be predictive of severe subcutaneous fibrosis. On haplotype analysis, the cumulative risk of developing severe fibrosis was observed in patients carrying both haplotypes of variant Homozygous AA genotype of ERCC4 Ex8 (1244G>A, rs1800067) and Homozygous TT genotype of XRCC5 (1401 G>T, rs828907) (p=0.010*, OR 26.340, 95% CI 4.014-76.568).

**Conclusion:**

We demonstrated significant associations between single nucleotide polymorphisms of DNA repair genes and radiation-induced subcutaneous fibrosis in patients of oropharyngeal carcinoma treated with radiotherapy. We propose to incorporate these genetic markers into predictive models for identifying patients genetically predisposed to the development of radiation-induced fibrosis, thus guiding personalized treatment protocols.

## Introduction

India has the highest incidence rate of oropharyngeal carcinoma (OPC) in the world, with majority (68.6%) of the patients presenting in locoregionally advanced stages of the disease (1). Radiotherapy with concurrent chemotherapy has been the standard non-surgical treatment for locally advanced OPC (2). However the toxicity of intensive treatment regimens also contributes to a substantial increase in patient morbidity and mortality, especially in developing countries like India, with patient profiles distinct from the western world. Most patients present with poor performance and nutritional status and inadequate support systems. This leads to poor compliance and treatment tolerability and hence, poor disease outcomes. Hence, majority of our patients receive definitive radiotherapy alone with conventional or altered fractionation schedules in order to achieve acceptable outcomes with minimum morbidity. Moreover, the enormous patient load in high volume referral centers imparts greater logistic difficulties in devoting the time and infrastructure to execute conformal treatment planning for every patient (3–5). Therefore, 2-dimensional conventional radiotherapy continues to be used for a significant proportion of our patients (6, 7).

The treatment fields used in conventional radiotherapy for OPC include large volumes of the oral cavity, pharynx and the neck resulting in high predisposition to radiation induced normal tissue toxicity (8). While acute radiotoxicities interrupt the routine treatment schedule and limit the radiation dose, long-term radiotoxicities significantly impair the quality of life of these patients (5). The most frequently encountered acute radiotoxicities in OPC are oral mucositis, dermatitis and dysphagia; while delayed toxicities include late-onset xerostomia, fibrosis and rarely, osteoradionecrosis of the mandible (9, 10).

Radiation-induced subcutaneous fibrosis, a late radiotoxicity response, results from dysregulation of inflammation and regeneration. It is one of the most common long-term toxicities of head and neck cancer (HNC) therapy and has been reported in more than 70% of the patients at some point after HNC treatment, causing cosmetic and functional impairment that significantly impacts quality of life (11–13). A number of factors increase the risk of radiation-induced fibrosis. These factors are treatment related (total dose, dose per fraction, volume irradiated, irradiation site and dose inhomogeneity, additional treatment like use of concomitant chemotherapy or surgery) or patient-specific (age, smoking, alcohol and tobacco usage and co-morbid conditions such as diabetes, vascular and connective tissue disorders) (11, 12).

However even with uniform treatment protocols, not all patients develop subcutaneous fibrosis and other radiotoxicities of the same severity. Apart from patient-specific factors, almost 80% of this inter-individual variability has been attributed to genetic differences amongst individuals (14, 15). The genetic pathways involved in radiation response(s) encompass a multitude of genes involved in processes such as DNA double-strand break (DSB) repair, DNA damage response, cell-cycle control, apoptosis, cellular antioxidant defenses and fibrosis (14–16).

Single nucleotide polymorphisms (SNPs) are DNA sequence variations that arise when a single nucleotide within a gene is altered. SNPs constitute more than 99% of all genetic variations that can affect mRNA stability, rates of transcription, protein translation and/or regulation of gene methylation resulting in dysregulated function and varying degrees of clinical radiosensitivity (14, 17). After a thorough literature search for identifying candidate genetic polymorphisms, we selected 7 genes related to DNA repair and one from the pro-fibrotic pathway for their presumed or demonstrated role in radiosensitivity. We hypothesize that SNPs in one or more genes involved in the above radiation response pathways can interfere with their function and trigger the development of radiotherapy induced normal tissue toxicity (16).

A multitude of DNA repair pathways are activated in response to radiation induced DNA damage. DNA double strand break (DSB) repair pathways include Homologous Recombination (HR) and Non-Homologous End Joining (NHEJ) repair. These are of critical importance in the repair of DNA damage that occurs in normal tissue adjoining the tumour as a result of radiation therapy. Single-stranded breaks (SSB) are repaired by Base Excision Repair (BER), Nucleotide excision repair (NER) and Mismatch repair (MMR) pathways. SNPs in DNA repair genes may alter the ability of these cells to repair radiation induced DNA damage ultimately resulting in more severe toxicity (18–20).

XRCC1 i.e. X-Ray repair cross complimenting 1 protein participates in BER pathway of SSB caused by ionizing radiation. XRCC1 (rs25487) polymorphism is a G to A transition at codon 399 that results in change from Arg to Gln within the XRCC1 protein. The resultant protein has altered fidelity and DNA repair efficiency. Besides, carriers of XRCC1 AA genotypes have higher levels of chromosomal breaks per cell when compared with other genotypes. Genetic variants of this gene have previously been linked to worse treatment outcomes as well as increased acute and late radiotoxicity (7, 21–23).

XRCC3 gene, a member of Rad-51-related genes, is an indispensable component of the HR pathway of DNA DSB repair and inter-strand cross-links, which plays an essential role in maintaining genomic stability. Variants of XRCC3 have been shown to be positively associated with late radiation-induced toxicity and elevated cancer risk (23–25).

The ERCC4 i.e. excision repair cross-complimentary group 4 gene forms a complex with ERCC1 to encode the two subunits of the ERCC1-XPF (xeroderma pigmentosum complementation group F) nuclease. This enzyme plays a central role in NER, DNA cross-link repair and is also involved in the incision step of NHEJ repair pathway (26). ERCC4 variants have been tested for their role in influencing radiation toxicities in HNCs (27).

XRCC5 is another important component playing a crucial role in NHEJ pathway of DNA DSB repair. SNPs in XRCC5 result in major structural changes in XRCC5 protein, rendering it unavailable for the NHEJ pathway. Polymorphisms in this gene have been shown to influence cancer risk and chromosomal radiosensitivity (28, 29).

Rad51 (RecA homolog, Escherichia coli) protein is a component of the HR repair pathway of DNA DSBs and inter-strand cross-links. Genetic variants of Rad51 influence mRNA stability and translational efficiency and have been linked to carcinogenesis and radiosensitivity (30–32).

TGFβ1 ie. transforming growth factor β1 encodes for the versatile cytokine TGFβ1 assumed to be involved in response to tissue injuries and has been suggested to play a role in radiation response. Polymorphic variations in TGFβ1 gene can alter protein expression contributing to the initiation, development, and persistence of radiation-induced fibrosis (33, 34).

The association of genetic polymorphisms with late radiotoxicities has been well explored in patients of breast and prostate cancer. However, few studies have explored the correlation between genetic polymorphisms and late radiotherapy toxicity in patients with HNCs (33, 35–38). Moreover, no such studies have been conducted on the Indian population, which harbors the largest number of HNC patients in the world, contributing to significant cancer-related morbidity (1).

Therefore, we conducted a prospective study on a carefully selected homogeneous cohort of OPC receiving definitive radical radiotherapy by two-dimensional conventional technique to evaluate the impact of SNPs of DNA repair and pro-fibrotic pathway genes on the severity of radiation-induced subcutaneous fibrosis.

Through this radiogenomic study, we aim to identify genetic biomarkers which can be incorporated into predictive models of radiation-induced subcutaneous fibrosis. This could aid in formulation of tailored treatment regimens by identifying ‘at-risk’ patient groups and assigning them to treatment by more conformal radiotherapy techniques like 3-dimensional conformal (3DCRT) or Intensity Modulated Radiation Therapy (IMRT). This personalization would also allow judicious allocation of the limited available resources and help achieve better outcome with minimum morbidity.

## Materials and Methods

This prospective study was conducted at a tertiary care referral center in North India with approval from the Institutional Ethics Committee (INT/IEC/2016/2124). Patients of newly diagnosed early inoperable and locoregionally advanced squamous cell carcinoma of the oropharynx (AJCC 7^th^ edition) being treated with two-dimensional radical radiotherapy were enrolled for a total period of two years and seven months. Patients who had undergone surgery or were receiving concurrent chemotherapy were excluded from our study. Those suffering from comorbidities such as diabetes mellitus, collagen vascular or immunosuppressive disorders were also excluded. Written informed consent was obtained from all study participants.

### Genotyping Analysis

5ml blood samples were drawn in Ethylenediaminetetraacetic acid (EDTA) vials from all recruited patients on the day of start of therapy and stored at -20 °C. For polymorphism analysis, DNA isolation was done using the Macherey Nagel DNA isolation kit™ (GmBH, Germany) according to manufacturer’s instructions.

Eight SNPs of seven DNA repair genes namely, XRCC1 (1196 G>A, rs25487), XRCC3 (722 C>T, rs861539), XRCC4 (-1394 T>G, rs689366), XRCC5 (-1401 G>T, rs828907), XRCC6 (-1310 C>G, rs22677437), ERCC4Ex11 (2505 T>C, rs1799801), ERCC4Ex8 (1244 G>A, rs1800067), Rad51 (172 G>T, rs1801321) and two SNPs of the pro-fibrotic pathway gene i.e. TGFβ1 (869 T>C, rs1982073) and TGFβ1 (-509 C>T, rs1800469) were analyzed by PCR (Polymerase Chain Reaction) RFLP (Restriction Fragment Length Polymorphism) (**Supplementary Data, Table 1** for PCR conditions and primer list).

### Treatment and Evaluation During Radiotherapy

All patients underwent pre-treatment simulation in a fluoroscopy simulator with an immobilizing thermoplastic cast and were treated by 2-dimensional conventional planning in a telecobalt unit or low energy Linear Accelerator as per established protocols at our center. Elective nodal irradiation was performed in all patients. Bilateral parallel-opposed lateral fields were used without any tissue compensators. An additional lower anterior field was used in selected patients.

A dose of 40 Gy in 20 fractions was delivered to the primary and draining lymph nodes over 4 weeks (phase I), which was followed by a dose of 20 Gy in 10 fractions after sparing the spinal cord (phase II). An additional 6 Gy in 3 fractions (phase III) was delivered to the gross tumour with 2 cm margins to a total dose of 66 Gy in 33 fractions. Dose schedule of 45Gy in 25 fractions over 5 weeks along with a concomitant boost of 22.5 Gy in 15 fractions to the gross primary and nodal disease with 1.5 to 2 cm margin over the last 3 weeks of treatment was used in selected patients. During treatment, patients were evaluated twice a week for acute radiation toxicities like oral mucositis, dysphagia and dermatitis.

### Follow-up and Toxicity Assessment

The patients were followed up at 6 weeks post completion of radiotherapy for assessment of response and toxicity evaluation, and every 3 months thereafter. The median follow up was 16 months (range 13-48 months). Patients with a follow-up of less than 12 months were excluded. The time of development of subcutaneous fibrosis was documented. The grade of subcutaneous fibrosis was jointly evaluated by two participating physicians according to the Radiation Therapy Oncology Group (RTOG) and European Organization for Research and Treatment of Cancer (EORTC) grading system (39). The maximum grade of fibrosis recorded over the length of the patients’ follow-up has been reported. For comparison, patients with severe fibrosis (≥G3), referred to as the radiosensitive group (cases), were compared to the patients with minor (≤G2) fibrotic reactions (controls).

### Patient-Specific Factors

In addition, patient-specific clinical characteristics such as age, smoking habits and history of tobacco chewing and alcohol consumption etc. were also documented and analyzed in relation to the severity of subcutaneous fibrosis.

### Statistical Analysis

The distribution of SNP genotypes and clinical characteristics within the radiosensitive group and the control group was analyzed by using Pearson’s Chi-square test or Fisher’s exact test. Binary logistic regression analysis was performed to evaluate the association of the significant variables with the risk of developing severe radiation-induced subcutaneous fibrosis. The genotypic frequencies were examined by estimating the Odd’s Ratio (OR) and 95% confidence interval (CI) of the wild, heterozygous and homozygous variant genotypes using the other two genotypes as the reference.

A multivariate logistic regression analysis was carried out to measure the independent predictive value of each SNP on the risk of severe subcutaneous fibrosis. A *p* value of <0.05 was considered statistically significant.

To explore the association of the combined effect of these variants with increased risk of severe fibrosis, a haplotype association analysis was performed for two polymorphisms; ERCC4Ex8 (1244 G>A, rs1800067) and XRCC5 (-1401 G>T, rs828907). Patients were subdivided into three risk categories. Group 1 was considered as the reference category and included patients with wild type homozygous and heterozygous genotypes of both polymorphisms. Group 2 included those patients who had haplotypes containing the variant homozygous genotype of any one of the polymorphisms indicating an intermediate risk category. Group 3 included those patients who had haplotypes containing the variant homozygous genotypes of both polymorphisms indicating a high-risk category. Binary logistic regression analysis was carried out to evaluate the risk associated with the latter two groups while keeping the first group as the reference.

All the above analyses were carried out with Statistical package for Social Sciences (SPSS) version 23.

## Results

### Study Patients

195 patients of OPC were enrolled in the study. Of these, 16 patients were excluded due to incomplete treatment and lack of follow-up. The remaining 179 patients were included for final analysis. Patient and disease characteristics have been listed in **Table 1**.

**Table 1:** Patient and disease characteristics.

Mean age (years)	58.9 ± 8.6
	N (%)
**Gender**	
Male Female	163 (89.4%) 16 (8.9%)
**Subsite**	
Base of tongue Soft palate Tonsil Vallecula	77 (42.5%) 59 (32.6%) 32 (17.7%) 13 (7.2%)
**Stage (WHO 7^th^ edition)**	
I II III IVA IVB	2 (1.1%) 21 (11.6%) 59 (32.6%) 95 (52.5%) 2 (1.1%)
**Smoking**	160 (89.4%)
**Tobacco chewing**	28 (17.2%)
**Alcohol intake**	104 (58.1%)

### Toxicity Analysis and Association With Genotypic and Clinical Factors

The genotypic frequencies of all SNPs and their association with radiation-induced subcutaneous fibrosis are shown in **Table 2**.

**Table 2:** SNP distribution and toxicity status.

SNP distribution	Fibrosis N (%)	
**XRCC1 (rs25487)**	≤Grade 2	Grade 3
Homozygous GG Heterozygous GA Homozygous AA	53 (73.6%) 65 (83.3%) 25 (86.2%)	19 (26.4%) 13 (16.7%) 4 (13.8%)
*p* value (Chi-square)	0.112	
**XRCC3 (rs861539)**	≤Grade 2	Grade 3
Homozygous CC Heterozygous CT Homozygous TT	75 (76.5%) 51 (82.3%) 2 (10.5%)	23 (23.5%) 11 (17.7%) 17 (89.5%)
*p* value	0.012*	
**XRCC4 (rs6869366)**	≤Grade 2	Grade 3
Homozygous TT Heterozygous TG Homozygous GG	105 (82.7%) 32 (71.1%) 3 (85.7%)	22 (17.3%) 13 (28.9%) 1 (14.3%)
*p* value	0.570	
**XRCC5 (rs828907)**	≤Grade 2	Grade 3
Homozygous GG Heterozygous GT Homozygous TT	42 (79.2%) 71 (82.6%) 10 (25%)	11 (20.8%) 15 (17.4%) 30 (75%)
*p* value	0.046*	
**XRCC6 (rs2267437)**	≤Grade 2	Grade 3
Homozygous CC Heterozygous CG Homozygous GG	69 (79.3%) 57 (81.4%) 17 (77.3%)	18 (20.7%) 13 (18.6%) 5 (22.7%)
*p* value	0.885	
**ERCC4 (rs1799801)**	≤Grade 2	Grade 3
Homozygous TT Heterozygous TC Homozygous CC	34 (75.6%) 59 (79.7%) 50 (83.3%)	11 (24.4%) 15 (20.3%) 10 (16.7)
*p* value	0.133	
**ERCC4 (rs1800067)**	≤Grade 2	Grade 3
Homozygous GG Heterozygous GA Homozygous AA	127 (81.4%) 16 (80%) 0 (0%)	29 (18.6%) 4 (20%) 3 (100%)
*p* value	0.003**	
**RAD51 (rs1801321)**	≤Grade 2	Grade 3
Homozygous GG Heterozygous GT Homozygous TT	55 (83.3%) 43 (78.2%) 45 (77.6%)	11 (16.7%) 12 (21.8%) 13 (22.4%)
*p* value	0.747	
**TgFβ1 (rs1982073)**	≤Grade 2	Grade 3
Homozygous TT Heterozygous TC Homozygous CC	134 (81.7%) 6 (40%) 0 (0%)	30 (18.3%) 9 (60%) 0 (0%)
*p* value	0.045*	
**TgFβ1 (rs1800469)**	≤Grade 2	Grade 3
Homozygous GG Heterozygous GT Homozygous TT	128 (80%) 11 (73.3%) 4 (100%)	32 (20%) 4 (26.7%) 0 (0%)
*p* value	0.246	

Bold values indicate the statistically significant p values on Chi-square/Fischer exact test; *p values ≤ 0.05, **p values < 0.01.

### Subcutaneous Fibrosis

Subcutaneous fibrosis was seen in 168 (93.9%) patients, and was more evident in the neck than the face. Thirty-six (20.1%) patients developed grade 3 fibrosis. Grade 2 fibrosis was observed in 47 (26.3%) patients. Grade 1 fibrosis was the most common and was seen in 85 (47.5%) patients. Grade 4 fibrosis was not seen in any patient.

Chi-square analysis revealed significant association between the genotypic frequencies of XRCC3 (722 C>T, rs861539) (p=0.012*), ERCC4Ex8 (1244 G>A, rs1800067) (p=0.003**), XRCC5 (1401 G>T, rs828907) (p=0.046*) and TGFβ1 (869 T>C, rs1982073) (p=0.045*) polymorphisms and severe subcutaneous fibrosis. Amongst clinical factors, history of alcohol intake showed a significant correlation (p=0.017*) with fibrosis.

On univariate logistic regression analysis, following SNPs were found to be significantly associated with the risk of severe subcutaneous fibrosis; Homozygous CC genotype of XRCC3 (722 C>T, rs861539) (p=0.015*, OR 2.227, 95% CI 1.741-6.696), Homozygous AA genotype of ERCC4Ex8 (1244 G>A, rs1800067) (p=0.012**, OR 23.143, 95% CI 1.974-271.362), Homozygous TT genotype of XRCC5 (1401 G>T, rs828907) (p=0.038*, OR 3.064, 95% CI 1.063-8.835) and Heterozygous TC genotype of TGFβ1 (869 T>C, rs1982073) (p=0.020*, OR 4.606, 95% CI 1.272-16.674) along with history of alcohol intake (p=0.023*, OR 3.584, 95% CI 1.189-10.803).

On multivariate logistic regression analysis, only the first three polymorphisms remained statistically significant and were independent predictors of the risk of severe subcutaneous fibrosis; Homozygous CC genotype of XRCC3 (722 C>T, rs861539) (p=0.013*, OR 2.350, 95% CI 1.089-5.382), Homozygous AA genotype of ERCC4Ex8 (1244 G>A, rs1800067) (p=0.001**, OR 11.626, 95% CI 2.490-275.901) and Homozygous TT genotype of XRCC5 (1401 G>T, rs828907) (p=0.020*, OR 2.188, 95% CI 1.652-7.334) (**Table 3**).

**Table 3:** Multivariate analysis of genotypic variables and clinical characteristics with risk of subcutaneous fibrosis.

Genotypic/Clinical Variable	Multivariate Odds Ratio (95% CI)	*p* Value
Homozygous AA genotype of ERCC4 Ex8 1244G>A Homozygous CC genotype of XRCC3 722C>T Homozygous TT genotype of XRCC5 1401G>T Heterozygous CC genotype of TGFβ1 (869 T>C, rs1982073) Alcohol intake	2.350 (1.089-5.382) 11.626(2.490-275.901) 2.188 (1.652-7.334) 4.368 (0.976-19.540) 3.209 (0.966-10.668)	0.001** 0.013* 0.020* * 0.054* * 0.057*

Bold values indicate the statistically significant p values on multivariate analysis; *p values ≤ 0.05, **p values < 0.01.

#### Haplotype Analysis

Presence of either of the haplotypes ie. variant Homozygous AA genotype of ERCC4Ex8 (1244 G>A, rs1800067) or Homozygous TT genotype of XRCC5 (1401 G>T, rs828907) was associated with a significantly increased risk of severe fibrosis (p=0.050*, OR 2.837, 95% CI 1.317-5.212) when compared to carriers of the wild type or heterozygous variants. A cumulative increased risk of developing severe fibrosis was observed in the presence of both haplotypes (p=0.010*, OR 26.340, 95% CI 4.014-76.568).

## Discussion

This study was conducted to investigate the impact of genetic variants of DNA repair and pro-fibrotic pathway genes on the development of severe radiation-induced subcutaneous fibrosis in patients of OPC.

SNPs of three DNA repair genes; XRCC3 (722 C>T, rs861539), ERCC4Ex8 (1244 G>A, rs1800067) and XRCC5 (1401 G>T, rs828907) were shown to significantly increase the risk of developing severe radiation-induced subcutaneous fibrosis. In addition, haplotypes of ERCC4Ex8 (1244 G>A, rs1800067) and XRCC5 (1401 G>T, rs828907) polymorphisms had a highly significant combined predictive effect on the risk of severe fibrosis.

XRCC3 polymorphisms have been extensively tested for their association with radiotoxicities in a variety of cancers. De Ruyck et al. analyzed XRCC3 polymorphisms in cervical cancer samples and concluded that SNPs of XRCC3 are associated with an increased risk of late toxic effects after radiation (34). Andreassen et al. reported that Thr/Thr genotype in XRCC3 codon 241 correlated with an increased risk of subcutaneous fibrosis as well as telangiectasia in breast cancer (33). Another study by Damaraju et al. found significant univariate associations between late rectal or bladder toxicity and XRCC3 SNPs (38). XRCC3 722 C>T allele has also been associated with an increased risk of radiation-induced late xerostomia in nasopharyngeal cancer patients (25). The association of this polymorphism with radiation-induced subcutaneous fibrosis in HNCs has not been demonstrated till date.

XRCC5 rs1051677 (T>C) C allele has been shown to be associated with severe subcutaneous fibrosis in patients of nasopharyngeal carcinoma in a study by Alsbeih et al, though the authors could not replicate these findings in their multivariate analysis (29). In a study by Yin et al, women with AG/AA genotypes of XRCC5 rs3835 (G>A) were at increased risk of severe radiation pneumonitis (40).

The association of SNPs of ERCC4 with the risk of radiation-induced fibrosis has not been reported previously. Although, studies have investigated its correlation with other radiotoxicity end-points such as dysphagia and feeding tube dependence in patients of HNC. In a study of 130 patients of OPC treated with radiotherapy, Kornguth et al. studied the association of two SNPs in XPF/ERCC4 and long-term use of percutaneous feeding tube. The Homozygous AA genotype of ERCC4 Ex8 1244G>A was associated with a reduced need for feeding tube, but this association was not statistically significant. Although the wild Homozygous TT genotype of the second SNP ERCC4 Ex11 2505T>C showed a protective effect and was significantly associated with decreased long-term gastrostomy tube dependence (27). In our study the variant allele of ERCC4 Exon 8 was associated with an increased risk of severe subcutaneous fibrosis.

The heterozygous TC genotype of TGFβ1 (869 T>C, rs1982073) correlated with severe subcutaneous fibrosis on univariate analysis. However, no significant association could be seen on multivariate analysis. In a study by Alsbeih et al. on patients of nasopharyngeal carcinoma, it was seen that the wild-type allele of TGFβ 869 T>C contributed to the severity of radiation-induced subcutaneous fibrosis (29). In sites other than head and neck, TGFβ 869 T>C polymorphism has shown significant associations with the risk of radiation-induced fibrosis in patients of breast cancer after breast conserving surgery (41). Other published studies suggested that the variant C allele was the risk factor (33, 42).

Of all patient specific clinical factors that were analyzed, history of alcohol intake was significantly associated with the risk of developing severe subcutaneous fibrosis. However, it failed to show significance in multivariate analysis. Although there is convincing evidence that acetaldehyde, the first metabolite produced during alcohol degradation, is responsible for the carcinogenic effect of ethanol owing to its multiple mutagenic effects on DNA (43), no association with risk of radiation related toxicities has been demonstrated till date.

Our findings showed significant association between SNPs of DNA repair genes and risk of severe subcutaneous fibrosis in patients of OPC treated with radiotherapy. These are encouraging results and suggest that genetic variations contribute to the severity of normal tissue toxicities after radiotherapy. More importantly, we also performed a haplotype association analysis of two polymorphisms for predicting the combined risk of severe subcutaneous fibrosis. Haplotype-based analysis may offer better genetic information and help improve the detection of causal genetic variants when compared with single SNP-based analysis (44).

OPC is a heterogeneous population with varying natural history and disease course. To ensure homogeneity in radiation portals and eliminate any confounding related to previous surgical resection or administration of concurrent chemotherapy, we included only those patients of OPC who were being treated with definitive radiotherapy. Most patients had advanced disease at presentation or bulky midline tumours involving the base of tongue and soft palate. Hence, bilateral neck irradiation was given in all cases, removing any confounding due to differences in field size (45, 46).

Though tumor HPV status is a strong and independent prognostic factor for survival among patients with OPC (47), it was not analyzed in our study. This was due to lack of adequate infrastructure along with lower HPV prevalence amidst the high tobacco burden in the country (48, 49). Likewise, approximately 90% of patients in our study population were smokers.

It is acknowledged that conformal techniques were not used in this study owing to the enormous patient load in a limited resource setting (3, 4). The number of patients with OPC treated at our center in the previous five years (2015–2019) ranged from 300-350 per year, as per the Hospital Based Cancer Registry data.

A candidate gene approach was used and only a limited number of SNPs were selected for study. SNPs represent a majority of heritable genetic variations, are often inherited together and multiple such variations may affect radiation response. Thus, the selection of candidate genes is a critical step in determining the genetic basis of normal tissue radiosensitivity. In this study, we have opted to give a high priority to SNPs that have been demonstrated to significantly influence biological processes such as DNA repair, which continue to be the most studied pathways for HNC outcomes (50).

The major limitation of a candidate gene approach is that it requires a prior knowledge of the gene function and previously unknown genetic variants involved in the phenotype are missed. Moreover, candidate gene studies are usually underpowered to detect the small effect sizes that are attributed to SNPs. It is critical to employ a genome-wide approach to overcome this limitation. Genome Wide Association Studies (GWAS) allow us to map the entire genome for the presence of genetic variants that could possibly have a significant impact on normal tissue radiosensivity. GWAS offer the advantage of studying all SNPs, including those in regulatory regions whose function is not fully understood. However, these studies require large sample sizes to be considered reliable and may detect many false positive SNPs that are unimportant in relevant biological processes. Replication studies should be carried out to distinguish the true positive SNPs that may have a role in influencing radiosensitivity (17). Also, other pathways that could be hypothetically involved in normal tissue radiosensitivity, such as oxidative stress response, activation of cell cycle checkpoints, inflammation and apoptosis are yet to be thoroughly investigated (29).

The results from this study, upon further validation would enable us to identify patients who are genetically predisposed to the development of severe radiation-induced subcutaneous fibrosis. We propose to incorporate these genetic markers into predictive models of normal tissue toxicity in combination with patient and clinical factors. Such a profile could divide patients into subgroups with different probabilities of developing toxicity, to permit irradiation up to the normal tissue tolerance for each subgroup. These ‘at risk’ patient groups could then be offered treatment with individualized protocols and with more conformal radiotherapy techniques like Intensity Modulated Radiation Therapy (IMRT). This is expected to aid in judicious allocation of the limited available resources in developing countries and also allow improved compliance with standard treatment schedules leading to better outcome with least morbidity.

## Conclusion

The ultimate goal of radiogenomics research is to tailor radiation therapy protocols based on a combination of genetic, clinical and treatment related factors, in order to optimize tumour control while causing minimal normal tissue damage. In the present study, we demonstrated significant associations between SNPs of DNA repair genes and severe radiation-induced subcutaneous fibrosis in oropharyngeal carcinoma. A multivariate predictive model was developed and combination of haplotypes were identified to characterize patients at high risk of severe subcutaneous fibrosis. The identified predictors of radiosensitivity are aimed to ultimately contribute to an algorithm for guiding therapy tailored to the patient’s risk and benefit profile.

## Data Availability Statement

The original contributions presented in the study are included in the article/**Supplementary Material**. Further inquiries can be directed to the corresponding author.

## Ethics Statement

The studies involving human participants were reviewed and approved by Institute Ethics Committee, Post Graduate Institute of Medical Education and Research, Chandigarh, India. The patients/participants provided their written informed consent to participate in this study.

## Authors Contributions

Study concept: SG and AP. Study design: AP. Data acquisition: AG and DM. Quality control of data and algorithms: AG, DM, and AP. Data analysis and interpretation: DM and AP. Statistical analysis: DM and AP. Manuscript preparation: AG and SB Manuscript editing: SB, SG, and AP. All authors contributed to the article and approved the submitted version.

## Funding

The study was partially funded by Special Research Grant by PGIMER, Chandigarh.

## Conflict of Interest

The authors declare that the research was conducted in the absence of any commercial or financial relationships that could be construed as a potential conflict of interest.
