# Supplementary material for: Genetic Variants of DNA Repair Genes as Predictors of Radiation-Induced Subcutaneous Fibrosis in Oropharyngeal Carcinoma
Source: Front Oncol. 2021 May 17;11:652049. doi: 10.3389/fonc.2021.652049 (PMC8165303; doi:10.3389/fonc.2021.652049)
Supplement: Supplementary file 1 [file Table_1.docx]

| **S.No** | **Polymorphism** | **Primers used for PCR** | | **PCR conditions** | | | | | **Amplicon size** | **Restriction enzyme used for RFLP (**Manufacturer**)** | **Digestion pattern (allele)** |
| --- | --- | --- | --- | --- | --- | --- | --- | --- | --- | --- | --- |
|  |  | **Forward Primer** | **Reverse Primer** | **Initial Denaturation** | **40 cycles of** | | | **Final extension** |  |  |  |
|  |  |  |  |  | **Denaturation** | **Annealing** | **Extension** |  |  |  |  |
| **1** | **XRCC1 (rs25487)** | CCCCAAGTACAGCCAGGTCC | CCGCTCCTCTCAGTAGTCTG | 94˚Cfor 1min | 94˚Cfor 45sec | 64˚Cfor 30sec | 72˚Cfor 90sec | 72˚Cfor 10 min | 242bp | HpaII (Fermentas) | G- 149bp, 93bp  A-242bp |
| **2** | **XRCC3 (rs861539)** | GACACCTTGTTGGAGTGTGT | GTCTTCTCGATGGTTAGGCA | 94˚Cfor 1min | 94˚Cfor 45sec | 55˚Cfor 30sec | 72˚Cfor 90sec | 72˚Cfor 10 min | 358bp | NlaIII (NEB) | C-302bp, 56bp  T- 197bp, 105bp, 56bp |
| **3** | **XRCC4 (rs6869366)** | GATGCGAACTCAAAGATACTGA | TGTAAAGCCAGTACTCAAACTT | 94˚Cfor 1min | 94˚Cfor 45sec | 59˚Cfor 30sec | 72˚Cfor 90sec | 72˚Cfor 10 min | 300bp | HincII (NEB) | T- 300bp  G- 200bp, 100bp |
| **4** | **XRCC5 (rs828907)** | TGTTTCCCGGATAGCCACTT | TGTAGAGTCAGACATATTCAGAGGT | 94˚Cfor 1min | 94˚Cfor 45sec | 55˚Cfor 30sec | 72˚Cfor 90sec | 72˚Cfor 10 min | 397bp | BfaI (NEB) | G- 397bp  T- 211bp, 186bp |
| **5** | **XRCC6 (rs2267437)** | CACTCGGCTTTTCTTCCATC | CCGAATCTCTATCCGGTGAA | 94˚Cfor 1min | 94˚Cfor 45sec | 54˚Cfor 30sec | 72˚Cfor 90sec | 72˚Cfor 10 min | 212bp | HinP1I (NEB) | C-212bp  G-135bp, 77bp |
| **6** | **ERCC4 (rs1799801)** | TCTGGTGCCCCTCTCCTCATG | CGGCAGTTTTTGGCATTCACC | 94˚Cfor 1min | 94˚Cfor 45sec | 64˚Cfor 30sec | 72˚Cfor 90sec | 72˚Cfor 10 min | 190bp | AlwNI (NEB) | T- 190bp  C-95bp |
| **7** | **ERCC4 (rs1800067)** | TCGGGTGAAGGAATAAGGGG | AGCCCGTTCTTTGTTTTGGG | 94˚Cfor 1min | 94˚Cfor 45sec | 56˚Cfor 30sec | 72˚Cfor 90sec | 72˚Cfor 10 min | 303bp | Xmn I (NEB) | G- 186bp, 117bp  A- 303bp |
| **8** | **RAD51 (rs1801321)** | TGGGAACTGCAACTCATCTGG | GCTCCGACTTCACCCCGCCGG | 94˚Cfor 1min | 94˚Cfor 45sec | 65˚Cfor 30sec | 72˚Cfor 90sec | 72˚Cfor 10 min | 131bp | NgoMIV (NEB) | G- 110bp, 21bp  T- 131bp |
| **9** | **TgFβ1 (rs1982073)** | CAAGCAGCATTTTATGTGGA | CAGTTGTTCTAGGTCGTAAC | 94˚Cfor 1min | 94˚Cfor 45sec | 56˚Cfor 30sec | 72˚Cfor 90sec | 72˚Cfor 10min | 202bp | NlaIII (NEB) | T- 103bp, 99bp  C- 202bp |
| **10** | **TgFβ1 (rs1800469)** | CCCGGCTCCATTTCCAGGTG | GGTCACCAGAGAAAGAGGAC | 94˚Cfor 5min | 94˚Cfor 45sec | 60˚Cfor 30sec | 72˚Cfor 60sec | 72˚Cfor 10 min | 808bp | Eco81I (NEB) | T-808 bp  C- 617bp, 191bp |

**Supplementary table 1: -** Polymorphisms analyzed, primer list and PCR conditions
